# ED diagnosis of peritoneal carcinomatosis

**DOI:** 10.1007/s10140-024-02238-w

**Published:** 2024-06-05

**Authors:** Devorah Scheinfeld, Carly Schwartz, Adam Z. Fink

**Affiliations:** 1 Montefiore Medical Center, Department of Radiology, 111 East 210 Street, Bronx, NY 10467 USA; 2 Department of Radiology, Division of Emergency Radiology, Montefiore Medical Center, Albert Einstein College of Medicine, 111 East 210 Street, Bronx, NY 10467 USA

**Keywords:** Peritoneal carcinomatosis, Ascites, Peritoneal malignancy, Emergency department

## Abstract

**Purpose:**

The goal of our study was to better characterize new CT diagnoses of peritoneal carcinomatosis (PC) in the ED, and to evaluate how to best identify the primary lesion. Prompt identification of the source of the carcinomatosis may allow for the patient to receive early initial care from the correct clinical service.

**Methods:**

All new CT cases of PC-like appearance identified on CT in the ED from January 2017 through July 2020. Each report and corresponding medical record were manually reviewed. Patient demographics, presence/absence of intravenous contrast, source organ predicted by the radiologist in the CT scan report, pathologic diagnosis, and amount of ascites were tabulated. Chi-tests were used to test the statistical significance of differences between groups.

**Results:**

Of the 131 CT cases of new PC-like appearance which received workup, 108 cases had pathologically proven PC and 23 cases had no underlying malignancy yielding a positive predictive value for actual PC of 82%. The most common cause of new PC in women was gynecological (66%), and in men was of GI tract origin (57%). Concordance between radiologist prediction and final pathology was higher with intravenous contrast (58%) compared to without contrast (40%); although this difference was not statistically significant (p = 0.19). A moderate or large amount of ascites was found in more than half of GYN primaries and in adenocarcinoma of unknown primary and there was a statistically significant difference in amount of ascites between cancer primaries (p = 0.01).

**Conclusion:**

A PC-like appearance on CT in the ED will likely be in patients with known malignancy, but of the new cases, there is a high PPV for it to represent new peritoneal carcinomatosis. Gynecological and GI malignancies are the most common cause in women and men, respectively, and this may help in focusing the radiologist’s search pattern. Usage of intravenous contrast may help in identifying a primary lesion, and the presence of high-volume ascites should suggest a GYN primary or adenocarcinoma of unknown primary when there is no other obvious primary lesion.

## Introduction

Peritoneal carcinomatosis refers to the spread of cancer cells to the peritoneum, which forms the lining of the abdominal (or peritoneal) cavity. Cancer cells may be adherent to the parietal peritoneum, the visceral peritoneum which lies against the outer surface of bowel and solid organs, the mesentery, and/or the omentum [1]. Technically, peritoneal carcinomatosis refers to peritoneal metastases from a malignancy of epithelial origin, while the terms peritoneal sarcomatosis and peritoneal lymphomatosis refer to metastases from tumors of mesodermal origin and lymphoid origin, respectively. However, given the lower prevalence of peritoneal sarcomatosis and lymphomatosis compared to carcinomatosis, as well as the overlapping imaging features of these entities, peritoneal sarcomatosis and lymphomatosis have been included under the category of peritoneal carcinomatosis in this paper for simplicity.

Patients with peritoneal carcinomatosis receiving traditional care (systemic chemotherapy and symptom directed treatment such addressing bowel obstruction) have an average survival time of less than six months. However, more recent treatment techniques such as cytoreductive surgery and hyperthermic intraperitoneal chemotherapy (HIPEC) have been found to increase survival time [1, 2], and new immunotherapy drugs have the potential to further increase length of survival [3].

In the emergency department (ED), a new diagnosis of peritoneal carcinomatosis may be suggested for patients who demonstrate a carcinomatosis-like appearance on abdominal/pelvic CT scan. CT findings consistent with a carcinomatosis-like appearance include peritoneal thickening, hyperenhancement, nodularity or masses, omental haziness, and ascites [4]. Suggesting this diagnosis on CT scan is of paramount importance, as this will trigger the necessary diagnostic workup to determine whether the findings truly represent peritoneal carcinomatosis, and appropriate cytological and histological testing can be undertaken to determine the primary tumor. Even at this early ED stage in the care of the patient, an accurate prediction on CT of the likely site of origin may be beneficial in directing the patient to the clinical service best suited to the workup and treatment of the underlying disorder (e.g. to medical oncology or gynecologic oncology).

The goals of our study were threefold. First, to determine the positive predictive value for peritoneal carcinomatosis in the setting of carcinomatosis-like appearance on CT scan in the ED. Second, to determine the incidence of various primary lesions in new cases of peritoneal carcinomatosis detected in the ED, in order to direct the radiologist as to which organs and areas to scrutinize most carefully when there is no obvious primary lesion. Third, to determine the accuracy of predicting the primary lesion on CT scan, and to assess whether administration of intravenous contrast or the amount of ascites may help with this prediction.

## Methods

IRB approval was obtained for this retrospective study performed at an urban academic medical center with four emergency department (ED) sites. To identify all true positive cases of peritoneal carcinomatosis detected using CT in the ED, the radiology department’s report database was searched for all CT scans imaging the abdomen from January 2017 through July 2020, with final reports containing the keywords: “carcinomatosis” or “caking” or “peritoneal metastasis” or “peritoneal metastases” or “peritoneal metastatic disease”. Each report was then reviewed manually. Cases of pulmonary lymphangitic carcinomatosis were excluded. Cases of non-carcinomatosis (such as when the report stated “carcinomatosis is considered unlikely”) were excluded as they were hedges which were typically ignored by the clinical service. As the goal was to identify new cases of peritoneal carcinomatosis, cases involving a patient with any known malignancy receiving follow up imaging in the ED were also excluded. Lastly, of the new cases of carcinomatosis-like appearance, those which did not receive further workup (for example, if the patient died precipitously or refused further workup), those for which the workup revealed an alternative diagnosis, and those for which the workup did not elucidate a diagnosis were excluded from the true positive cohort.

For each patient from the true positive study cohort, the following data were recorded: patient demographics, presence/absence of intravenous contrast, source organ predicted by the radiologist in the CT scan report, pathologist provided diagnosis (from the medical record), and amount of ascites (none, mild, moderate, large). If the radiologist offered no prediction of the source organ or provided a differential in the CT scan report, the case was recorded as “radiologically indeterminate for a primary lesion”. The exception to this was if the radiologist predicted “ovarian or uterine origin”, this was recorded as being of GYN origin and was considered concordant with the pathology results if the source was uterine or ovarian in origin. When the report provided a range of ascites, such as “mild-to-moderate” ascites, the higher amount (i.e. moderate) was used for purposes of our analysis. Chi-tests were used to test the statistical significance of differences between groups. All data were tabulated and statistical analysis was performed using Microsoft Excel (Microsoft, Redmond, WA).

## Results

**Fig. 1:**
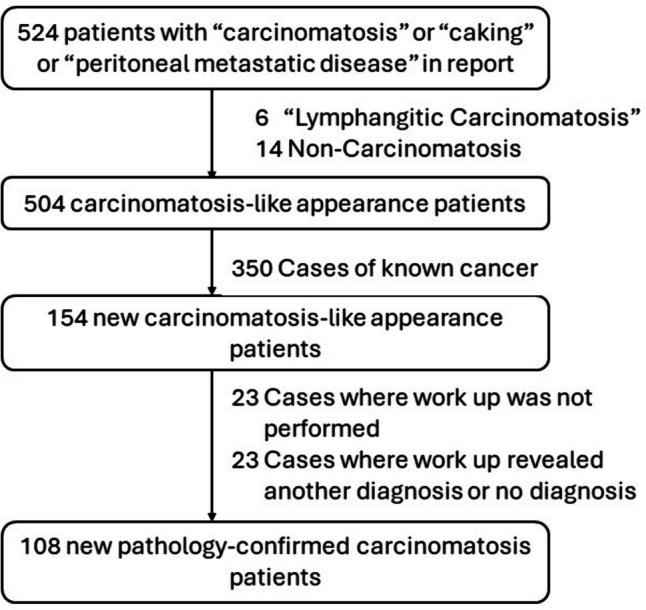
Patient flow in this study describing how the final cohort of 108 patients was gathered

**Fig. 2:**
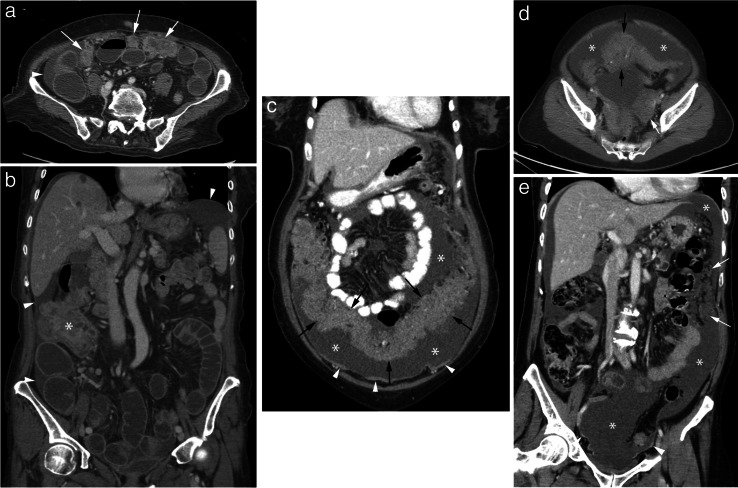
Case examples of carcinomatosis. a,b Axial and coronal CT images from a 61-year-old woman demonstrates centrally necrotic peritoneal nodules (arrows) and ascites (arrowheads) consistent with peritoneal carcinomatosis. The primary lesion was pathologically confirmed to be from the ascending colon (asterisk). c,d Coronal and axial CT images from a 54-year-old woman demonstrates a large quantity of ascites (asterisks), thick omental caking (black arrows) and plaque-like peritoneal thickening (arrowheads). The primary lesion was pathologically confirmed to be from the left ovary (white arrow). e Coronal CT image from a 58-year-old woman with adenocarcinoma of unknown primary. Note the large amount of ascites (asterisks), omental nodularity (arrows) and nodular and plaque-like peritoneal thickening (arrowheads). A primary lesion was not identified in this patient

Five hundred twenty-four CT scan reports met the report database search criteria (Fig. 1). Six reports referred to pulmonary lymphangitic carcinomatosis and 14 referred to non-carcinomatosis and were excluded. Of the remaining 504 carcinomatosis-like appearance cases, 350 cases involved patients with known malignancy and were excluded, yielding 154 cases of new carcinomatosis-like appearance. Of these 154 cases, 23 cases did not receive further workup. Of the remaining 131 positive CT cases for which further workup was performed, 23 revealed no underlying malignancy (i.e. 23 false positive cases). These consisted of 11 patients who underwent biopsy which revealed no cancer as well as 12 patients who did not undergo biopsy and had (average ± SD) 5.9 ± 1.1 years (range 4.1–7.3 years) of follow up which did not show cancer. These 23 false positive cases were reviewed and found to have alternative diagnoses including peritoneal TB, endometriosis, pancreatic ascites, or no ultimate diagnosis. Therefore, the final true positive cohort included 108 cases of pathologically proven peritoneal carcinomatosis (Fig. 2).

For purposes of analysis, new cases of pathologically proven carcinomatosis were divided into the following categories based upon site of the primary tumor: GYN origin (includes cervical, uterine, endometrial, fallopian tube, ovarian, and Mullerian tumors), Pancreaticobiliary (includes pancreatic, biliary, and gallbladder tumors), Adenocarcinoma of unknown primary, Proximal GI (includes esophageal, gastric, and small bowel tumors), Colon, Hepatocellular carcinoma, and Other (includes a single case each of desmoplastic small round cell tumor, prostate cancer, small bowel lymphoma, and lymphoma) (Table 1).

**Table 1:** Pathologically proven causes of peritoneal carcinomatosis in the 108 cases in the true positive cohort

Origin of Primary Tumor on pathology	Female		Male		Combined	
GYN*	51	66%	NA	NA	51	48%
Pancreaticobiliary**	8	10%	5	17%	13	12%
Adenocarcinoma of unknown primary	7	9%	3	10%	10	9%
Proximal GI***	6	8%	8	27%	14	13%
Colon	5	6%	9	30%	14	13%
Hepatocellular carcinoma	1	1%	1	3%	2	2%
Other****	0	0%	4	13%	4	4%
Total	78		30		108	

^*^GYN includes cervical, uterine, endometrial, fallopian tube, ovarian, and Mullerian tumors

^**^Pancreaticobiliary includes pancreatic, biliary, and gallbladder tumors

^***^Proximal GI includes esophageal, gastric, and small bowel tumors

^****^Other includes a single case each of desmoplastic small round cell tumor, prostate cancer, small bowel lymphoma, and lymphoma

The number and percentage of cases are tabulated

In the true positive case cohort, 78/108 patients (72%) were female, and the mean patient age ± standard deviation was 64.9 ± 14.4 (range 17–89 years). Cases were read by a total of 33 radiologists with a mean ± standard deviation of 3.3 ± 3.1 cases (range 1–16 cases) per radiologist. All cases were read by staff attending radiologists, and none by fellows acting as attendings for on-call coverage. 102 CT scans were performed using GE LightSpeed VCT 64-slice MDCT scanners (GE Healthcare, Milwaukee, WI), and 6 CT scans were performed using a GE Optima 540 16-Slice MDCT scanner (GE Healthcare, Milwaukee, WI).

Of the 131 new cases with a carcinomatosis-like appearance on CT that underwent further workup, the positive predictive value of new carcinomatosis-like appearance was 82%. The frequency of various primary tumors and the concordance between the radiologist’s prediction of primary tumor and the pathologist’s diagnosis are tabulated in Table 2. Of the CT exams for the 108 true positive cases, 93 (86%) were performed with intravenous contrast. The diagnosis was concordant in 56% of cases overall, with higher rates when the exam was performed with intravenous contrast (54/93 cases, 58%) compared to without intravenous contrast (6/15 cases, 40%); however, this difference was not statistically significant (p = 0.19). The amount of ascites on initial ED scan varied by primary lesion type, with a moderate or large amount of ascites found in more than half of GYN primaries and in adenocarcinoma of unknown primary (Table 3). The difference in amount of ascites between cancer primaries was statistically significant (p = 0.01).

**Table 2:** Pathologically proven causes of peritoneal carcinomatosis in the 108 cases in the true positive cohort, along with concordance between the radiologist’s prediction of origin of primary tumor and the pathologist’s diagnosis

	# of Cases	Concordant cases	Concordant case %
GYN	51	29	57%
Proximal GI	14	10	71%
Colon	14	8	57%
Pancreaticobiliary	13	11	85%
Adenocarcinoma of unknown primary	10	0	0%
Other	4	1	25%
Hepatocellular carcinoma	2	1	50%
Total	108	60	56%

**Table 3:** Amount of ascites categorized by primary tumor

	No or mild ascites	Moderate or large ascites	% with moderate or large ascites
GYN	16	35	69%
Proximal GI	9	5	36%
Pancreaticobiliary	9	4	31%
Colon	9	5	36%
Adenocarcinoma of unknown primary	2	8	80%
Other	3	1	25%
Hepatocellular carcinoma	1	1	50%

Greater than half of cases of GYN cancer and adenocarcinoma of unknown primary had a moderate or large amount of ascites on CT scan

## Discussion

As new and more effective treatments for peritoneal carcinomatosis emerge, patients are beginning to live longer with the disease. Thus, more patients may present to the ED with complications of their underlying cancer or from their treatment. Indeed, in our initial database of 524 cases with a carcinomatosis-like appearance on CT, 350 patients (67%) had a known cancer (Fig. 1). When imaging for presurgical planning and follow-up, structured reporting utilizing the peritoneal carcinomatosis index (PCI) (which divides the peritoneum into 13 locations including a three-by-three grid over the abdomen and pelvis, upper and lower jejunum, and upper and lower ileum) and necessitating mention of the largest implant sizes and presence of vascular and ureteral involvement has been advocated [5, 6]. A comprehensive description such as this is generally not possible in the ED setting.

The goal of our study was to evaluate new cases of peritoneal carcinomatosis diagnosed in the ED, in patients without a preexisting malignancy. This diagnosis is typically made on abdominal CT scan, the workhorse of ED abdominal imaging. Other modalities can be used to diagnose or characterize peritoneal carcinomatosis [7], but these are infrequently performed in the ED setting.

In our study, the most common cause of new peritoneal carcinomatosis in women was of GYN origin (66%), and in men was of GI tract origin (proximal GI plus colon, 57%) (Table 1). Our study sample did not find any cases of peritoneal mesothelioma, a rare primary peritoneal tumor [8]. Among all cases of new carcinomatosis-like appearance on CT which received further workup, the positive predictive value (PPV) of pathologically confirmed new peritoneal malignancy was 82%. Many mimics of peritoneal carcinomatosis have been described in the literature, including peritoneal tuberculosis (TB) [9], fibroids [10], peritoneal sarcoid [11], endometriosis [12], actinomycosis [14], and splenosis [13], some of which were found to result in false positive CT findings in our study. The PPV of these mimics will vary based on their respective prevalences in the population. In our study, only one in 131 patients (0.8%) with a new carcinomatosis-like appearance who received further workup was diagnosed with peritoneal TB, which is not unexpected given the low prevalence of TB in the United States [14]. CT findings suggestive of peritoneal TB rather than carcinomatosis include loculated ascites, splenomegaly, and lymph node conglomeration, while focal hepatic and nodular omental lesions favor carcinomatosis [11]. A multivariate model [15] and texture analysis [16] have also been used to differentiate these two entities.

Identifying the primary lesion on CT scan once cancer has spread to the peritoneum is challenging but is beneficial in the ED setting in order to direct the trajectory of care to the correct medical service. The use of IV contrast may assist in this identification. For example, IV contrast may help delineate a hypo- or hyper-enhancing parenchymal primary lesion which would be obscured on a non-contrast study, particularly in the setting of ascites and fluid infiltration of abdominal fat (which is commonly present in peritoneal carcinomatosis). In our study, the primary lesion was identified in 40% of cases without IV contrast compared with 58% of cases with IV contrast, suggesting the utility of IV contrast; however, this difference was not statistically significant. Ascites is believed to develop in peritoneal carcinomatosis due to lymphatic obstruction preventing the resorption of peritoneal fluid, as well as due to secretion of vascular permeability factor by the cancer cells [1]. We found a statistically significant association of higher volume ascites with GYN tumors and adenocarcinomas of unknown primary compared to other primary tumors, although overlap was present (Table 3).

Our study had several limitations. Due to the retrospective design and reliance on a report database keyword search, there may have been positive cases which were not included in our cohort because the interpreting radiologist did not appreciate the presence of peritoneal carcinomatosis. This could have been a problem particularly for CT scans without intravenous contrast, as the presence of ascites can easily obscure peritoneal nodularity on non-contrast exams. We also did not analyze differences in accuracy of reporting amongst radiologists of different subspecialties (e.g. body radiologists, ED radiologists, and radiologists on-call reading outside their subspecialty). Because all radiologists in our department routinely interpret body CT while on-call, we believe that this reflects a typical ED practice setting where there is a baseline level of competence amongst all the radiologists, with some having varying levels of advanced expertise. Finally, our finding of intravenous contrast improving prediction accuracy was not statistically significant. However, intravenous contrast has been shown to improve lesion detection in other contexts [17], and it is therefore likely to also improve diagnostic performance in the context of peritoneal carcinomatosis. A larger cohort would probably be necessary to achieve statistical significance.

In conclusion, most cases of carcinomatosis-like appearance encountered on CT scan in the ED setting will be in patients with known malignancy, but of the new cases, there is a high PPV for carcinomatosis-like appearance to represent new peritoneal carcinomatosis rather than other non-cancer diagnoses. GYN malignancies were the most common primary lesion in women and GI primaries were the most common in men. The presence of high-volume ascites suggests a GYN primary or adenocarcinoma of unknown primary. In our cohort, usage of intravenous contrast improved accurate prediction of the primary lesion from 40 to 59%; however, this was not statistically significant. Future studies with a larger cohort size may be helpful in determining the role of intravenous contrast in accurately predicting the location of the primary lesion.

## Data Availability

Upon request.
